# The first presentation of a case of nail-patella syndrome newly diagnosed at the onset of rheumatoid arthritis: a case report

**DOI:** 10.1186/s12891-024-07242-2

**Published:** 2024-02-14

**Authors:** Kazuya Matsumoto, Yoshinori Matsumoto, Shoichi Nawachi, Yosuke Asano, Yu Katayama, Yoshia Miyawaki, Takayuki Katsuyama, Eri Katsuyama, Yoshihisa Nasu, Ken-Ei Sada, Jun Wada

**Affiliations:** 1 Department of Nephrology, Rheumatology, Endocrinology and Metabolism, Faculty of Medicine, Dentistry and Pharmaceutical Sciences, Okayama University, 2-5-1 Shikata-Cho, Kita-Ku, Okayama, 700-8558 Japan; 2 Department of Rheumatology, Chugoku Central Hospital, 148-13 Kamiiwanari, Miyuki-Cho, Fukuyama, 720-0001 Japan; 3 Department of Medical Laboratory Science, Okayama University Graduate School of Health Sciences, 2-5-1 Shikata-Cho, Kita-Ku, Okayama, 700-8558 Japan; 4 Department of Orthopaedic Surgery, Faculty of Medicine, Dentistry and Pharmaceutical Sciences, Okayama University, 2-5-1 Shikata-Cho, Kita-Ku, Okayama, 700-8558 Japan; 5 Department of Clinical Epidemiology, Kochi Medical School, Kochi University, Kohasu, Oko-Cho, Nankoku, 783-8505 Japan

**Keywords:** Nail-patella syndrome, Rheumatoid arthritis, Joint dislocation, Iliac horn, Case report

## Abstract

**Background:**

Nail-patella syndrome (NPS) is a rare autosomal dominant disorder that is characterized by dysplasia of the nails, hypoplasia and/or dislocation of the patella and the presence of iliac horns. Using the CARE guidelines, we present the first reported case of NPS that was newly diagnosed at the onset of rheumatoid arthritis (RA).

**Case presentation:**

A 74-year-old man was admitted to our hospital due to an 8-month history of arthralgia in bilateral wrists, elbows and fingers. He had a past history of glaucoma and left patella dislocation that had been operatively recentered at the age of 15 years. Laboratory data showed elevated levels of serum C-reactive protein and rheumatoid factor and an elevated titer of anti-SS-A antibodies, while estimated glomerular filtration rate (eGFR), titers of other antibodies and the results of a urinary test were normal. An X-ray showed deformity of bilateral radial heads and the right elbow, and magnetic resonance imaging (MRI) of his hands showed synovitis and erosion in the multiple swollen joints of the wrists and fingers. In addition to these typical features of RA, he had bilateral thumb nail dysplasia with mild hypoplasia of bilateral patellae and iliac horns as shown by the X-ray. He was diagnosed as having autosomal dominant disorder NPS co-existing with RA and he was treated with methotrexate in combination with an oral Janus kinase (JAK) inhibitor, leading to induction of remission.

**Conclusions:**

We have presented a rare case of NPS that was newly diagnosed at the onset of RA. Clinical and radiographic findings of NPS are highlighted in this case report for diagnosing NPS on the basis of typical manifestations.

## Key messages

Nail-patella syndrome was newly diagnosed on the basis of typical clinical and radiographic findings at the onset of rheumatoid arthritis.

## Background

Nail-patella syndrome (NPS) is a rare autosomal dominant systemic disorder that is characterized by dysplasia of the nails, hypoplasia, aplasia or dislocation of the patella, and the presence of iliac horns [1, 2]. NPS is often associated with the presence of multiple organ involvement including glomerulopathy, glaucoma, gastrointestinal dysfunction and neuropathy [1, 2]. In addition to these clinical features, a heterozygous loss of function pathogenic mutation in the *LMX1B* gene, which is located on the long arm of chromosome 9 in the 9q34.1 locus and encodes LIM homeobox transcription factor 1-beta (LMX1B), is identified in about 95% of patients with NPS [1, 2].

Rheumatoid arthritis (RA) is an autoimmune disorder of the joints characterized by inflammatory arthritis and subsequent joint destruction [3]. NPS and RA share some musculoskeletal symptoms such as swan-neck deformities of the fingers and joint dislocation [1, 4, 5], and the prevalences of NPS and RA are 1/50000 and 1/100–1/200 [6–8], respectively, while no case of NPS co-existing with RA has been reported.

Here, using the CARE guidelines, we report the first case of NPS that was diagnosed at the onset of RA presenting synovitis.

### Case presentation

**Fig. 1:**
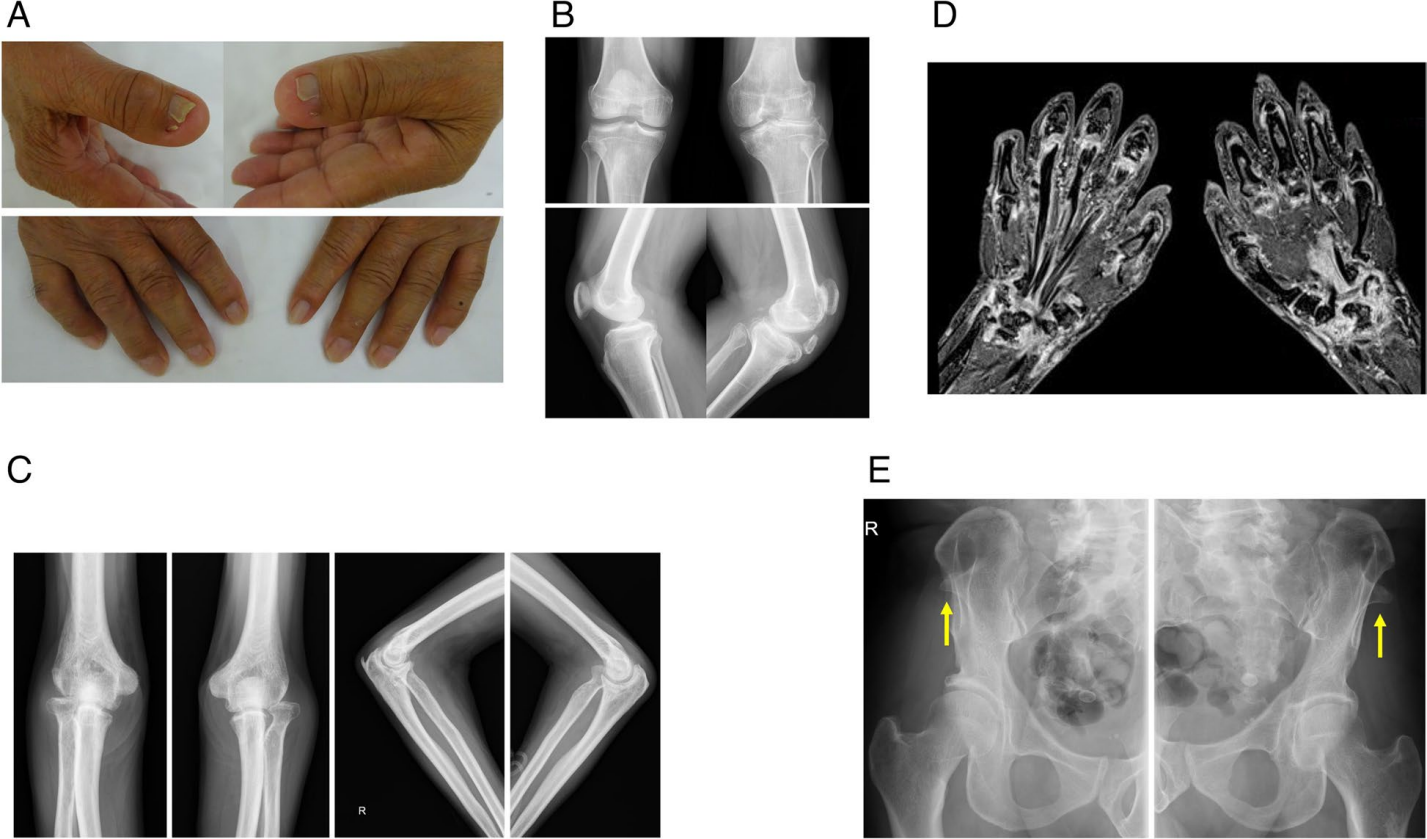
**A** Images of the patient’s nails. Dysplasia on the ulnar border of bilateral thumb nails (upper) and triangular lunules (lower). **B** X-ray of the knees showed mild hypoplasia of bilateral patellae and degeneration of the left knee. **C** X-ray of the elbows showed hypoplasia of the radial head and joint space narrowing in the right elbow. **D** MRI of the hands showed synovitis with synovial hyperplasia, bone marrow edema and erosion in bilateral wrists and in proximal interphalangeal and metacarpal phalangeal joints. **E** X-ray of the pelvis showed bilateral iliac horns (arrow)

A 74-year-old man was admitted to our hospital due to an 8-month history of arthralgia without any family history. He had a past history of glaucoma and benign prostatic hyperplasia in addition to left patella dislocation that had been operatively recentered at the age of 15 years. On admission, his body temperature was 37.2 °C and other vital signs were normal. Physical examination revealed multiple swollen joints, including bilateral wrist, metacarpophalangeal and proximal interphalangeal joints, bilateral elbows and right shoulder. Additionally, he showed dysplasia of bilateral thumb nails on the ulnar border and triangular lunules from birth, which were also observed in his sister (Fig. 1A, upper and lower). Laboratory data showed elevated levels of serum C-reactive protein and rheumatoid factor and an elevated titer of anti-SS-A antibodies, while estimated glomerular filtration rate (eGFR), titers of serum complements, anti-cyclic citrullinated peptide antibodies (CCP), anti-nuclear antibodies (ANA), anti-double stranded DNA antibodies (dsDNA) and myeloperoxidase (MPO)/proteinase 3 (PR3)-anti-neutrophil cytoplasmic antibodies (ANCA), and the results of a urinary test were normal (Table 1). X-rays of the joints showed deformity of bilateral radial heads and the right elbow, mild hypoplasia of bilateral patellae (height/width/thickness (mm): 35.2/34.8/15.6 (right) and 38.0/39.9/10.6 (left), respectively, [9]) and the right humeral capitellum and radius, and degenerative change of the left knee without evidence of destruction of the feet (Figs. 1B, 1C). Magnetic resonance imaging (MRI) of his hands showed synovitis with synovial hyperplasia, bone marrow edema and erosion in bilateral wrists, proximal interphalangeal and metacarpal phalangeal joints (Fig. 1D). In addition to these findings that were consistent with RA, an X-ray of his pelvis showed bilateral iliac horns, which are rarely observed in RA (Fig. 1E).

**Table 1:** Laboratory findings on admission

**Hematological test**						**Serological test**		
WBC	5,260	/µL	Na	139	mmol/L	RF	94.4	U/mL
Hb	15.8	g/dL	Cl	106	mmol/L	Anti-CCP Ab	0.5	U/mL
Plt	188	× 10^3^/µL	K	3.6	mmol/L	ANA	1280	fold
			UA	5.4	mg/dL	(Speckled pattern)		
**Biochemical test**			CK	47	U/L	Anti-SS-A Ab	> 240	U/mL
TP	8.1	g/dL	CRP	0.69	mg/dL	Anti-SS-B Ab	0.99	U/mL
Alb	3.7	g/dL	C3	110	mg/dL	Anti-dsDNA Ab	1.4	IU/mL
AST	19	U/L	C4	17.2	mg/dL	Anti-RNP Ab	2.15	U/mL
ALT	13	U/L	HbA1c	5.9	%	Anti-ARS Ab	< 0.50	index
ALP	86	U/L	KL-6	313	U/mL	MPO-ANCA	< 0.50	IU/mL
γ-GTP	27	U/L	Ferritin	227	ng/mL	PR3-ANCA	0.7	IU/mL
LDH	195	U/L	ESR	16	mm/hr			
BUN	14.5	mg/dL	MMP-3	178.5	ng/mL	**Urinalysis**		
Cr	0.55	mg/dL	IgG	2640.4	mg/dL	pH	6.0	
eGFR	108.5	mL/min /1.73m^2^				Protein	( ±)	
						Occult blood	( ±)	
						Glucose	(-)	

*Alb* Albumin, *ALP* Alkaline phosphatase, *ALT* Alanine aminotransferase, *ANA* Anti-nuclear-antibody, *Anti-ARS Ab* Anti-aminoacyl tRNA synthetase antibody, *Anti-CCP Ab* Anti-cyclic citrullinated peptide antibody, *Anti-dsDNA Ab* Anti-double stranded DNA antibody, *Anti-RNP Ab* Anti-ribonucleoprotein antibody, *AST* Aspartate aminotransferase, *BUN* Blood urea nitrogen, *CK*: Creatine kinase, *Cr* Creatinine, *CRP* C-reactive protein, *eGFR* Estimated glomerular filtration rate, *ESR* Erythrocyte sedimentation rate, *γ-GTP* Gamma-glutamyl transpeptidase, *Hb* Hemoglobin, *IgG* Immunoglobulin G, *KL-6* Krebs von den Lungen-6, *LDH* Lactate dehydrogenase, *MMP-3* Matrix metalloprotease 3, *MPO-ANCA* Myeloperoxidase antineutrophil cytoplasmic antibody, *Plt* Platelets, *PR3-ANCA* Proteinase-3 anti-neutrophil cytoplasmic antibody, *RF* Rheumatoid factor, *TP* Total protein, *UA* Serum uric acid, *WBC* White blood cells

In view of these physical and radiographic findings including nail deformity that was also observed in his sister, patella hypoplasia, iliac horns and a history of dislocation of the left knee, we finally made a diagnosis of NPS in addition to RA presenting articular synovitis. Additionally, lip biopsy examination disclosed Sjogren’ syndrome (focus score = 3) without sicca syndrome.

He was treated with oral methotrexate (MTX) in combination with a biologic drug, which failed to improve his condition and was replaced with an oral Janus kinase (JAK) inhibitor, and he achieved remission.

## Discussion

We present a previously undescribed case of NPS that was not diagnosed in the patient until the age of 74 years and was newly diagnosed at the onset of RA. NPS shows various musculoskeletal symptoms including hypoplasia, aplasia and/or dislocation of patellae, iliac horns, talipes and lumbar lordosis and scoliosis. Arthralgia of finger joints and reduction of flexion of the distal interphalangeal joints co-existing with hyperextension of proximal interphalangeal joints that results in swan-neck deformity [1] are also observed in NPS [10] (Table 2).

**Table 2:** Clinical features and frequencies in this case, NPS and rheumatoid arthritis. NA: not applicable

	Present case	Rheumatoid arthritis	Nail-patella syndrome (NPS)	Frequency in NPS	Ref
Arthralgia	Yes	Yes	Yes	NA	NA
Nail deformity	Yes	Longitudinal or ridging clubbing	Absent, hypoplastic or dystrophic Longitudinal ridging, pitting, discolored or separating triangular lunules	98%	[1, 11]
Knee involvement	Yes	Inflammatory arthritis with bone erosion	Hypoplasia Aplasia Subluxation Dislocation	74%	[1, 3]
Iliac horn	Yes	No	Yes	70–80%	[6]
Swan-neck deformity	No	Yes	Yes	58%	[1]
Morning stiffness	Yes	Yes	No	NA	[3]
Eye involvement	Yes (glaucoma)	Scleritis Uveitis Primary open-angle glaucoma	Primary open-angle glaucoma	9.6%	[1, 12]
Renal involvement	No	No	Various abnormality	NA	[5]
Proteinuria	No	No	Yes	21%	[4]
Microscopic hematuria	No	No	Yes	18%	[4]

Additionally, NPS shows systemic manifestations including numbness, open-angle glaucoma and glomerulonephritis with proteinuria and hematuria [1, 4], and renal involvement is the first manifestation that occurs in 30–50% of NPS patients [2]. Reduction of sensation to pain and temperature in the hands and feet in NPS is due to impairment of the connection of Aδ and C fibers with interneurons in the dorsal spinal cord [13]. Although clinical diagnostic criteria for NPS have not been established, NPS is generally diagnosed by clinical and radiographic manifestations, and nail deformity and iliac horns are especially considered pathognomonic [6].

In addition to these clinical features, a heterozygous loss of function pathogenic mutation in the *LMX1B* gene is identified in about 95% of patients with NPS [1, 2]. LMX1B is a transcription factor that contains two N-terminal zinc-binding LIM domains, 1 homeodomain, and a C-terminal glutamine-rich domain and is essential for the normal development of dorsal limb structures, the anterior segment of the eye, the glomerular basement membrane, and serotonergic and dopaminergic neurons [1, 2]. Mice lacking Lmx1b, a murine orthologue of LMX1B, display ventral-ventral distal limbs with abnormal kidney, eye and cerebellar development and with lack of nails, patella, and distal ulna and have a hypoplastic scapula and bent clavicle that are phenotypic similarities to human NPS [14–16]. In addition to mutations in the *Lmx1b* gene in mice, two conserved Lmx1b-associated cis-regulatory modules (LARM1 and LARM2), which are enhancers of *Lmx1b* and elevate *Lmx1b* expression, are necessary for Lmx1b-mediated limb dorsalization [17]. Loss-of-function variations in LARM1/2 genes have been identified in NPS patients with a normal LMX1B coding sequence [17]. Some of the musculoskeletal manifestations observed in NPS are common in RA, while knockdown of LMX1B has been reported to have a protective effect on osteoarthritis [18], and a genetic link between LMX1B and RA has not been reported. Although we did not perform a genetic test since the patient did not agree with the test, the physical and radiographic findings as well as dysplasia of bilateral thumb nails of both the patient and his sister were conclusively consistent with the diagnosis of NPS.

The prognosis of NPS is dependent on its organ involvement. Bongers et al. reported that patients with an *LMX1B* mutation located in the homeodomain showed significantly more frequent proteinuria and higher values of proteinuria than those in subjects carrying mutations in the LIM domains [4], indicating that the risk of developing nephropathy is dependent on the location of variants in the *LMX1B* gene. Fifteen percent of NPS patients progress to end-stage renal disease, suggesting that renal involvement is a critical prognostic factor [19]. Angiotensin II receptor antagonists and angiotensin-converting enzyme inhibitors have been proposed as treatment for patients with nephropathy [20], while no therapeutic strategy for NPS has been developed except for joint replacement surgery [21].

Although NPS is known to be a rare hereditary disease, the prevalences of NPS and RA are 1/50000 and 1/100–1/200 [6–8], respectively, and co-existence of NPS and RA could be observed in real-world clinical practice. In the present case, arthralgia and swollen joints were due to the development of RA, while joint deformity as well as dislocation of the patella may be only due to NPS (Table 2). The deformity observed in the patient’s right elbow was caused by a secondary change due to hypoplasia of the humeral capitellum and radius, not bone destruction due to RA, in view of the short-term history of RA, indicating that not only dislocation of the left patella but also deformity of the right elbow may be caused by NPS.

## Conclusions

We have presented a rare case of NPS that was newly diagnosed at the onset of RA. Clinical and radiographic findings of NPS are highlighted in this case report for diagnosing NPS on the basis of typical manifestations.

## Data Availability

The materials used during the current study are available from the corresponding author on reasonable request.

## Abbreviations

NPS: Nail-patella syndrome
RA: Rheumatoid arthritis
CCP: Cyclic citrullinated peptide
ANA: Anti-nuclear antibodies
dsDNA: Double stranded DNA
MPO: Myeloperoxidase
PR3: Proteinase 3
ANCA: Anti-neutrophil cytoplasmic antibodies
MRI: Magnetic resonance imaging
MTX: Methotrexate
JAK: Janus kinase
LMX1B: LIM homeobox transcription factor 1-beta
LARM: Lmx1b-associated cis-regulatory modules
